# Effects of Oral Care Intervention on Gingivitis-Mediated Induction of Nitric Oxide Synthesis and Oxidative Stress

**DOI:** 10.3390/ijms27135882

**Published:** 2026-06-30

**Authors:** Malgorzata Klukowska, Sancai Xie, Lijuan Li, Tom Huggins, Julie Ashe, Chelsea Tan, Benjamin Circello, Xingtao Wei, Niranjan Ramji, Donald J. White, Aaron R. Biesbrock

**Affiliations:** 1 Global Oral Care R&D, The Procter & Gamble Company, Mason Business and Innovation Center, 8700 Mason-Montgomery Road, Mason, OH 45040, USA; ramji.n@pg.com (N.R.); lamadental@outlook.com (D.J.W.); aaronbiesbrock@gmail.com (A.R.B.); 2 Corporate Functions R&D, The Procter & Gamble Company, Mason, OH 45040, USA; xie.s@pg.com (S.X.); li.l.28@pg.com (L.L.); adellcrocket@gmail.com (T.H.); ashe.j@pg.com (J.A.); circello.bt@pg.com (B.C.); wei.x.3@pg.com (X.W.); 3 Biosciences, Procter & Gamble International Operations SA, P&G Singapore Innovation Center, 70 Biopolis St., Singapore 138547, Singapore; tan.c@pg.com

**Keywords:** stannous fluoride, dentifrice, metabolomics, urea cycle, nitric oxide, citrulline, oxidative stress, gingivitis

## Abstract

This research examined host metabolic processes associated with gingivitis and potential impacts of interventions. First, gingival brush samples from a previously reported clinical study in which participants used an intervention (stannous fluoride (SnF_2_) toothpaste/cetylpyridinium chloride rinse/oscillating-rotating electric toothbrush) or control (standard fluoride toothpaste/manual toothbrush) were analyzed with Metabolon metabolomic assessment. Next, hypotheses were generated about mechanisms of metabolic observations and tested in two in vitro models. Human primary blood cells were treated with endotoxins and outer membrane vesicles (OMVs) in the presence of SnF_2_. In clinical brush samples, 46 metabolites decreased with intervention at Week 3, including 9- and 13-hydroxyoctadecadienoic acid, citrulline, adenine, and hypoxanthine. Seven metabolites related to nitrogen metabolism and citrulline production were selected for targeted quantification. They were low in health and increased in gingivitis. Targeted quantification demonstrated reduced citrulline, succinate, ornithine and deoxycarnitine (*p* ≤ 0.001 for all) between baseline and Week 6 with intervention; reductions were greater for intervention vs. control for citrulline, succinate and deoxycarnitine at Week 6 (*p* ≤ 0.019). In vitro, endotoxin and OMVs increased citrulline and nitric oxide (NO). SnF_2_ inhibited elevation of both. Citrulline, deoxycarnitine, succinate, and ornithine were identified as potential novel markers of gingivitis. Elevated NO production is a potential driver of oxidative stress, reduced with intervention.

## 1. Introduction

The global prevalence of gingivitis in adults is reported to exceed 75% [1,2,3,4,5,6,7]. Left untreated, gingival inflammation can progress to periodontitis, which includes loss of clinical attachment, recession, increased tooth mobility and tooth loss [8,9,10,11,12]. Periodontal infections and actions of microbes are associated with chronic inflammatory diseases, such as diabetes, rheumatoid arthritis, Alzheimer’s disease, and cardiovascular diseases [13,14,15,16,17]. Microbial communities in dental plaques produce or comprise a variety of ‘virulence factors’ that contribute to activation of the innate immune response and trigger inflammation [18]. Some virulence factors are associated with metabolism of bacteria in the dental plaque, including, for example, production and secretion of various enzymes (e.g., gingipains) [19,20] and various non-protein metabolites, such as volatile sulfur species [21] and short-chain carboxylic acids [22]. Other virulence factors are associated with the molecular composition of bacteria themselves, such as lipopolysaccharide (LPS), lipoteichoic acid (LTA) and peptidoglycans found in the cell walls of the microbes and secreted in outer membrane vesicles (OMVs) [23,24,25]. The LPS, LTA and peptidoglycan molecular structures of oral pathogens are “pathogen associated molecular patterns” (PAMPs). PAMPs are detected and recognized by specialized receptors called pattern recognition receptors (PRRs), which are ubiquitously expressed in numerous immune and epithelial cells [26,27]. The PRRs play a crucial role in the innate immune system to fight against a variety of microbial pathogens, including bacteria, viruses, parasites, fungi, and protozoa [26,27]. The best-known PRRs are the Toll-like receptors (*TLR*s). Different *TLR*s are expressed on distinct subsets of immune and non-immune cell types. Ten human *TLR*s have been characterized and designated *TLR1*-*TLR10*.

In the periodontium, *TLR*s have been detected in various leukocytes traversing the periodontal tissues, gingival epithelial cells, gingival fibroblasts, endothelium, periodontal ligament fibroblasts, osteoblasts, osteoclasts and cementoblasts [28]. Based on available evidence, *TLR4* and *TLR2* appear to be associated with development of inflammatory responses to oral pathogens and development of chronic inflammation associated with periodontal disease, with LPS (a component of the outer membrane of Gram-negative bacteria) recognized by *TLR4*, while peptidoglycan (a major component of various bacterial cell walls) is recognized by *TLR2* [28]. When microbial virulence factors bind to *TLR2* and *TLR4*, they trigger a signaling cascade that results in the production of cytokines, a signature of activating immune responses. Using gene signaling reporter assays, it has been demonstrated that both *TLR2* ligands (peptidoglycans and LTA) and *TLR4* ligands (LPS) are more abundant in the subgingival plaques of bleeding sites than in non-bleeding sites [29,30]. The higher abundance of LPS in the subgingival plaques of bleeding sites was confirmed using a Limulus Amebocyte Lysate assay [30].

Stannous fluoride (SnF_2_) dentifrice has been clinically proven to provide a broad range of hard tissue (e.g., caries, sensitivity) [31] and soft tissue benefits, namely inhibiting plaque and controlling gingivitis [32,33,34,35]. Previous mechanistic research has documented the impact of SnF_2_ dentifrice on virulence factors associated with the metabolic activity of dental plaque and its ability to directly interact with microbial PAMPs. Collectively, SnF_2_ dentifrice has been documented to: (1) modify and suppress plaque metabolism [36]; (2) reduce quorum sensing and obstruct biofilm formation [37]; (3) directly bind LPS and LTA from periodontal pathogens [38]; (4) modulate LPS-based activation of *TLR*s in multiple gene reporter cell lines, including HEK293 cells [39,40]; (5) suppress *Porphyromonas gingivals* (*P. gingivalis*) OMV-based *TLR* activation [40]; (6) interfere with *TLR* activation of expression of proinflammatory cytokines in human primary monocytes [39]; and (7) diffuse into and remain substantive in the subgingival sulci of gingivitis patients [41]. More recently, metabolomic investigations revealed that SnF_2_ dentifrice suppressed activation of central metabolism (glycolysis and citric acid cycle) [42]. These metabolic effects were demonstrated in oral lavage samples applying Metabolon’s global biochemical profiling mass spectrometry procedures [43]. Subsequent in vitro examinations confirmed the efficacy of SnF_2_ dentifrice in blocking adenosine triphosphate (ATP) activation in a novel ATP biosensor engineered cell line [42].

Since dentifrice is typically used in an oral hygiene routine with other products, a 6-week randomized, examiner-blind clinical study was conducted to evaluate the effects of an oral hygiene intervention including SnF_2_ dentifrice, a cetylpyridinium chloride (CPC) oral rinse, and an oscillating-rotating (OR) electric toothbrush compared to a control (standard 0.243% sodium fluoride (NaF) dentifrice and a soft manual toothbrush) on gingival bleeding among participants with gingivitis [44]. (The study has been presented at a conference, but to date it has not been published in a peer-reviewed journal. Study information can also be found on clinicaltrials.gov, NCT07211061, where it was retrospectively registered.) The oral hygiene intervention provided statistically significant reductions in gingival bleeding sites (9%, 21% and 54% lower at Weeks 1, 3 and 6, respectively; *p* < 0.001 for all) relative to the control. See Supplementary Table S1. Gingival brush samples were taken during the study for post hoc analysis employing Metabolon’s biochemical profiling.

The purpose of this research was to conduct metabolomic profiling of the gingival brush samples collected from the oral hygiene intervention group (SnF_2_ dentifrice, CPC rinse, and the OR electric toothbrush) and control group (NaF dentifrice and manual toothbrush) to identify host responses to the combined intervention [44]. Findings from these analyses led to additional hypotheses about a potential mechanism for SnF_2_ to contribute to the effects observed from the multi-agent intervention. The impact of SnF_2_ was then evaluated in subsequent complementary laboratory studies to drive plausibility that SnF_2_ alone could contribute to the proposed mechanism. Analyses of the gingival brush samples from the intervention clinical study and the follow-up in vitro evaluations of SnF_2_ alone are the basis for this report.

## 2. Results

**Figure 1:**
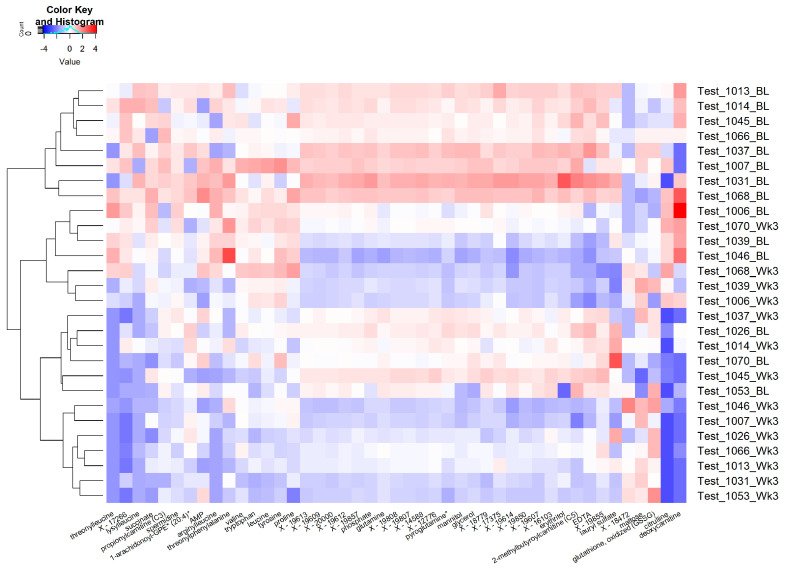
Changes in metabolite levels in gingival brush samples after 3 weeks of using an oral hygiene intervention. Heatmap illustrating the relative abundance of 46 prominent candidate metabolites (unadjusted *p* ≤ 0.05, corresponding q-value = 0.229) identified in response to the oral hygiene intervention. Individual *p* values, q values and statistical method are listed in Supplementary Table S2. Results show exploratory analyses of a subsample of 14 clinical trial participants in the intervention (test) group at Baseline and Week 3. * Pyroglutamine, also called pyroglutamate, is a cyclized derivative of the amino acid glutamic acid or glutamine. * 1-arachidonoyl-GPE (20:4) refers to 1-arachidonoyl-*sn*-glycero-3-phosphoethanolamine, a lysophosphatidylethanolamine containing an arachidonic acid acyl chain.

**Figure 2:**
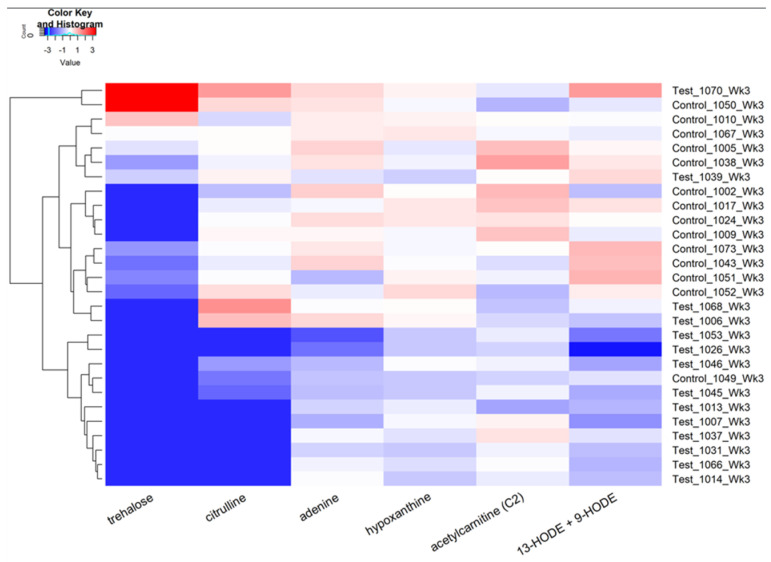
Comparison of metabolite levels in gingival brush samples following three-week use of oral hygiene intervention (test) or control. These candidates exhibited the strongest nominal differences between the intervention and control groups at Week 3. Samples from the control group predominantly clustered together, distinct from samples in the intervention group. This analysis represents an exploratory investigation using a subsample of 14 clinical trial participants in the control group and 14 participants in the intervention (test) group at Week 3. Individual *p*-values, FDR-corrected q-values, and statistical methods for each metabolite are detailed in Supplementary Table S3.

Brush-based sampling of gingival tissue was carried out at Baseline, Week 3, and Week 6. A random subset of Baseline and Week 3 samples was forwarded to Metabolon for analysis of metabolic changes in gingival tissue during this study. The metabolomic analysis identified 46 prominent candidate metabolites exhibiting a nominal (unadjusted) *p*-value ≤ 0.05 in response) to the oral hygiene intervention. These findings are limited by the small subsample; they are considered hypothesis-generating and warrant further investigation (Figure 1, Supplementary Table S2). Of note, a key metabolite, citrulline (unadjusted *p* = 0.041, q = 0.229), related to nitrogen metabolism, was reduced relative to Baseline in the intervention group following 3 weeks of use. Comparisons were also made between the intervention and control groups at 3 weeks (Figure 2). Citrulline (unadjusted *p* = 0.049, q = 0.252), 9-hydroxyoctadecadienoic acid (9-HODE), 13-hydroxyoctadecadienoic acid (13-HODE) (unadjusted *p* = 0.032, q = 0.208), adenine (unadjusted *p* = 0.007, q = 0.072) and hypoxanthine (unadjusted *p* = 0.005, q = 0.065) were all observed to be nominally decreased (unadjusted *p* ≤ 0.05) between the intervention and control at Week 3.

**Figure 3:**
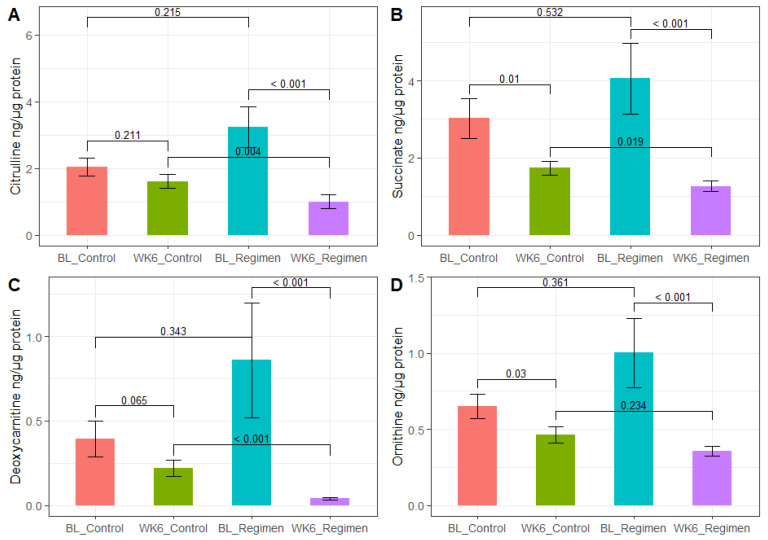
Changes in concentrations of selected metabolites in gingival brush samples from oral hygiene intervention (regimen) and control. Seven metabolites were selected based on the Metabolon profiling results. The brush samples were reanalyzed using 7-plex targeted analysis for samples collected at 6 weeks. Results were compared between Baseline and Week 6 in each group and compared between two groups. The four metabolites demonstrating a reduction in the intervention group are shown: (**A**) citrulline; (**B**) succinate; (**C**) deoxycarnitine; (**D**) ornithine. Baseline data show analyses of samples from 18 clinical trial participants in the control group and 15 participants in the intervention (regimen) group. Week 6 data reflect analyses of samples from 31 participants in the control group and 32 participants in the intervention (regimen) group.

Based on the results generated from the exploratory analysis performed on the Baseline and Week 3 subset, seven metabolites were selected for additional analysis. Six of these (citrulline, ornithine, arginine, malic acid, fumaric acid, and succinic acid) were selected based on their relationship to the NO biosynthesis pathway and the urea cycle. The last, deoxycarnitine, was previously detected in oral lavage samples [42], showing elevated levels in individuals with gingivitis. A targeted analysis for these specific metabolites was conducted on all Week 6 gingival brush samples and remaining Baseline samples. Results were compared between Baseline and Week 6 within each group and compared between the two groups. Results for the four metabolites showing a statistically significant reduction in the intervention group are illustrated in Figure 3. Citrulline, succinate, ornithine and deoxycarnitine were significantly reduced (*p* ≤ 0.001 for all) between Baseline and Week 6 in the intervention group. Citrulline, succinate and deoxycarnitine were also significantly reduced (*p* ≤ 0.031 for all) in the intervention group in comparison with the control at Week 6. The consistent observation of citrulline suppression observed in the test group clinical samples prompted the hypothesis that elevated levels of citrulline could be due to increased NO production and that one or more components of the intervention could be mitigating this phenomenon. This hypothesis was then directly tested in vitro.

**Figure 4:**
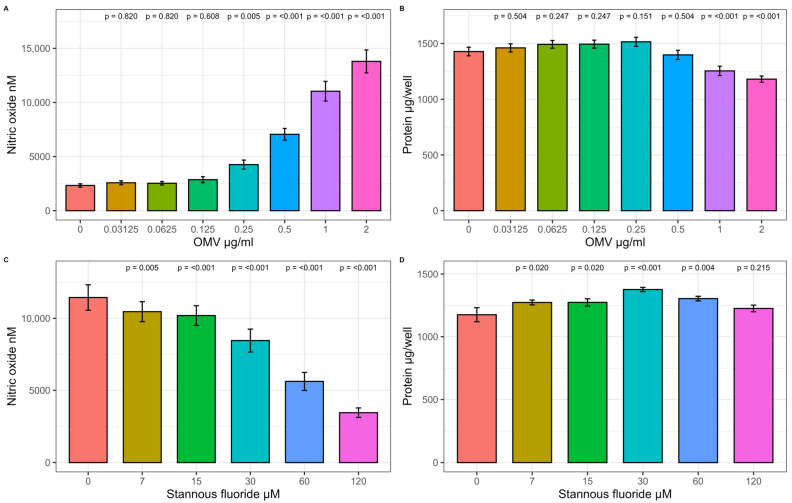
Stannous fluoride (SnF_2_) inhibited *Porphyromonas gingivalis (P. gingivalis)* outer membrane vesicle (OMV)-induced nitric oxide (NO) production in mouse macrophages. (**A**) OMVs (μg/mL) stimulated the production of NO in an increasing dose-dependent manner. (**B**) OMV dose response on cell viability; total cellular protein levels were maintained until 1 μg/mL. (**C**) Effects of SnF_2_ dose response, ranging from 7 to 120 μM, on the production of NO (nM) stimulated by 1 μg/mL *P. gingivalis* OMVs. (**D**) SnF_2_ at 7–120 μM maintained cellular protein levels. *p*-values indicate statistical testing versus 0 μg/mL (**A**,**B**) or 0 μM (**C**,**D**).

**Figure 5:**
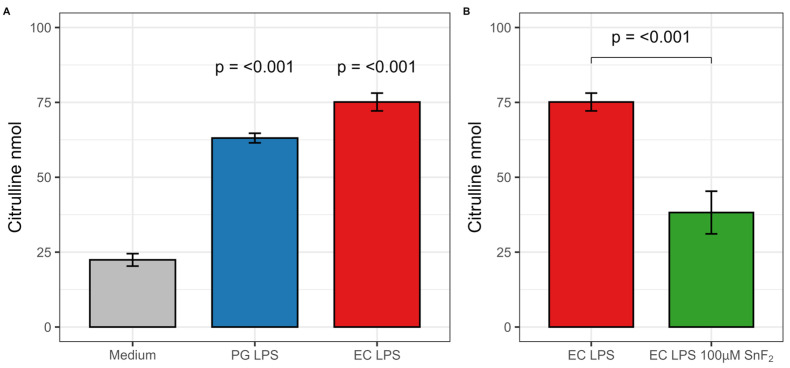
Citrulline production by primary human blood monocytes stimulated by lipopolysaccharides (LPS) from *Escherichia coli* (*E. Coli*) or *P. gingivalis*. Citrulline production was measured using a citrulline enzyme-linked immunosorbent assay (ELISA) kit. (**A**) LPS-stimulated citrulline formation. *p*-values above the bar indicate statistical testing versus Medium. (**B**) SnF_2_ (100 μM) addition to media suppressed citrulline stimulation. *p*-value indicates statistical testing versus control without SnF_2_.

In the first set of in vitro experiments, NO formation was induced by addition of *P. gingivalis* OMVs to a murine macrophage cell line following a protocol similar to that previously reported [40]. A murine macrophage cell line (RAW 264.7 cells) was selected to model OMV-stimulated NO production as a generalizable surrogate for human macrophage cell NO production. While human macrophages do produce NO to combat intracellular pathogens and modulate immune responses [45], the reduced production compared to RAW 264.7 cells would have made it more challenging to study NO formation in response to *P. gingivalis* OMVs. Instead, a widely used murine macrophage cell line (RAW 264.7 cells) was chosen to facilitate the investigation of NO formation induced by *P. gingivalis* OMVs following an established protocol [40]. The results are shown in Figure 4. OMVs stimulated NO formation in a dose-dependent manner, with significant increases beginning at 0.25 μg/mL OMV (*p* = 0.005 vs. 0 μg/mL OMV). SnF_2_ inhibited the effects of OMV in promoting NO formation in macrophages, with significant reductions beginning at 7 μm SnF_2_ (*p* = 0.005 vs. 0 μm SnF_2_). In the second set of in vitro experiments, citrulline formation was promoted in human monocytes by stimulation with LPS from either *Escherichia coli* (*E. coli*) or *P. gingivalis*. These data are arguably more clinically meaningful given the use of human monocytes. Results are shown in Figure 5. Both *E. coli* and *P. gingivalis* LPS stimulated citrulline formation at 100 ng/mL levels in incubation media. Addition of SnF_2_ to media significantly suppressed citrulline stimulation in human blood monocytes.

## 3. Discussion

This work was structured as a complementary investigation to a clinical study examining the impact of an oral health intervention on gingivitis. First, a profile of in vivo metabolic changes associated with intervention use was generated. That data was then used to generate a potential mechanistic avenue consistent with the observed results. This putative mechanism was then explicitly tested in vitro to support the contribution of one specific component of the intervention (SnF_2_) to the observed results. The overall data is consistent with the concept that gingivitis results in increased levels of NO in gingival tissue, which acts as an immediate source of oxidative stress. The elevation of NO is driven by excess LPS, which is a potent activator of monocytes and macrophages [46]. Exposure of macrophages to LPS results in activation of the Nuclear Factor-*κB* transcription factor, which orchestrates a gene expression program resulting in higher inducible NO synthase (*iNOS*) levels [47,48]. Elevated *iNOS* then substantially increases production of NO from arginine with the concomitant production of citrulline, which has potential utility as a convenient biomarker for this process in tissue samples.

In vivo, evidence for NO acting as a direct source of oxidative stress in gingivitis can be observed by the increased levels of adenine, hypoxanthine, and deoxycarnitine. These metabolites may accumulate after inactivation of the enzymes used to process them, which are damaged by NO or related free radicals. Xanthine oxidase is the enzyme responsible for the conversion of hypoxanthine to xanthine, and excess NO has been shown to react with superoxide to generate peroxynitrite and permanently inactive xanthine oxidase [49]. Similarly, NO can directly inactivate the carnitine/acetylcarnitine carrier protein resulting in cytosolic build-up of these compounds and subsequent metabolic backpressure [50].

The presence of succinate and 9- and 13-HODE as differential markers is potentially driven by an independent oxidative stress pathway, whereby gingivitis elevates activity of central metabolic pathways with a corresponding increase in superoxide levels [42]. The excess superoxide then reacts with linoleic acid to generate various racemic mixtures of 9-HODE and 13-HODE. These molecules are common markers of oxidative stress isomers in tissues undergoing inflammation [51]. 9-HODE and 13-HODE are also known to exert downstream proinflammatory effects [52,53]. Specifically, 9-HODE has been shown to upregulate Interleukin-6, Interleukin-8, and Granulocyte-Macrophage Colony-Stimulating Factor in keratinocytes, as well as Matrix Metalloproteinase-1.and Matrix Metalloproteinase-3.in fibroblasts [54]. Furthermore, 13-HODE has been reported to be an important inducer of Intercellular Adhesion Molecule 1 (*ICAM-1*) in endothelial cells, which is critical for regulating leukocyte recruitment from the underlying capillary beds to local tissue sites of inflammation [55]. Likewise, succinate is elevated in inflammation and sustains Interleukin -1β production through Hypoxia-Inducible Factor-1α stabilization [56]. Succinate activates immune cells via its receptor Succinate Receptor 1 and exacerbates disease [56,57]. Elevated succinate levels within the gut lumen have been reported to be associated with microbiome dysbiosis, as well as with inflammatory bowel disorder [58].

In vitro, both NO and citrulline levels increased following an endotoxin challenge. In these experiments, macrophages were stimulated by LPS-containing OMVs to produce NO through *iNOS* stimulation. SnF_2_ was able to suppress promotion of NO formation by macrophages. NO production by macrophages is a crucial part of the innate immune response to infection, and monocyte differentiation is stimulated by endotoxins. Hence, monocytes themselves are key targets for virulence stimulation during infection [59,60,61]. LPS from *E. coli* and *P. gingivalis* also stimulated citrulline production by human primary monocytes. SnF_2_ directly inhibited LPS-induced citrulline production by the monocytes in this assay. Importantly, the levels of SnF_2_ showing activity are below the stannous concentration found in subgingival crevicular fluid 30 min post-brushing with SnF_2_ toothpaste [41].

**Figure 6:**
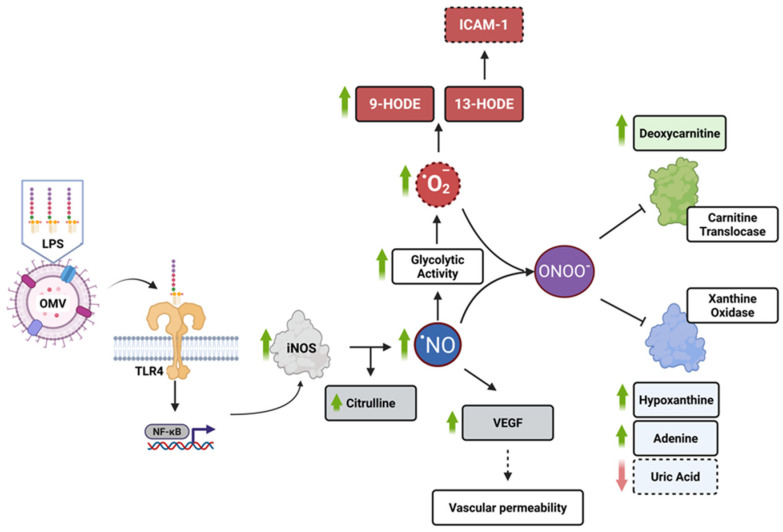
NO–citrulline on endothelial cells: Vascular inflammation and oxidative stresses. Green arrows indicate increased activity or levels, and red arrows indicate decreased activity or levels. Solid arrows indicate established or direct relationships/conversions, while dashed arrows indicate indirect or less direct relationships. Lines ending with a perpendicular bar indicate inhibition or suppression. Red boxes indicate inflammatory mediators, green boxes indicate metabolic products, grey boxes indicate enzymes, purple shapes indicate reactive oxygen/nitrogen species, and blue circle indicates nitric oxide. Dashed borders indicate hypothesized or inferred impacts/levels that were not directly measured or observed. Created in https://BioRender.com.

The observed results prompted the development of a proposed model for the potential effects of virulence factors on endothelial cells associated with NO and citrulline expression and associated processes in inflammation. This is illustrated in Figure 6. In the proposed model, virulence factors (e.g., OMV and/or LPS) stimulate NO and citrulline production by macrophages and monocytes. Over-production of NO leads to an increase in oxidative stress, both direct and indirect—as supported by the succinate, hypoxanthine, and deoxycarnitine increases observed in this study. The main target of NO is endothelial cells, and similarly, bacterial virulence is likewise known to stimulate production of vascular endothelial growth factor (*VEGF*) [62,63], whose target is endothelial cells. Two additional proinflammatory biomarkers that are associated with endothelial cells, *ICAM-1*—also known as Cluster of Differentiation 54—and Vascular Cell Adhesion Molecule 1 (*VCAM-1*), were previously shown to be increased in gingivitis, supporting this model [64,65].

The intervention treatment (SnF_2_ toothpaste, CPC rinse, and OR electric toothbrush) reduced NO expression and citrulline production, and, in principle, therefore could plausibly reduce *VEGF*, *ICAM-1* and *VCAM-1* production, thus decreasing vascular effects of inflammation and oxidative stresses on endothelial cells in the capillary beds of gingival connective tissue, although this idea needs to be experimentally tested and the specific role of SnF_2_ confirmed. Furthermore, the effects of SnF_2_ in vitro in suppressing inflammatory processes induced by virulent bacterial toxins in monocytes/macrophages can be speculated to be of value in reducing systemic burdens associated with periodontal infections. It is known that Gram-negative bacteria like *P. gingivalis* and *Treponema denticola* can spread throughout the body, promoting systemic inflammation [66,67,68,69]. The reactivity of SnF_2_ with bacterial endotoxin, including LPS or OMV comprising LPS, is hypothesized to suppress the reactivity of these virulence components with monocytes in local gingival tissues and the underlying capillary beds of the circulatory system, thereby inhibiting inflammatory conditions. Future research should be directed at further elucidating these hypotheses regarding this relationship and its local and systemic implications.

There are some potential limitations to this research. The clinical trial has not been published in a peer-reviewed journal to date, and it was not prospectively registered. However, it was conducted in 2011 when prospective registration was less common in dental research. The findings are based on post hoc subset analyses, but the collection of samples for analysis was prospectively outlined in the study protocol. The samples were analyzed three to six years after collection. Research suggests there is low risk of sample degradation with storage at −80 °C during this timeframe [70]. Any possible effects would have been balanced across groups. The alcohol–essential oil mouthrinse used as a single dose prior to each salivary sample collection has recognized anti-bacterial and anti-inflammatory properties when used under chronic dosing conditions, which may be a possible confounder with respect to the composition of saliva samples and also a potential limitation of this research. Importantly, this was controlled research comparing an oral hygiene intervention (SnF_2_ dentifrice, CPC mouthrinse, and OR electric toothbrush) to a control (NaF dentifrice and manual toothbrush). Both groups’ salivary sample collection was conducted under identical conditions, allowing for differences in observed outcomes to be solely attributed to the effects of the intervention and control. Furthermore, salivary metabolite profiles have been reported in the literature to be relatively unaffected by alcohol exposure, oral hygiene frequency, and the use of medications and supplements [71,72]. While the Metabolon platform employed for metabolic profiling is proprietary, all other aspects of the analyses are described in Section 4. Finally, the sample size of this exploratory work was modest, providing initial identification of the effects of the three-treatment intervention and the putative role of SnF_2_ as part of the intervention. Future clinical work with larger sample sizes evaluating SnF_2_ alone should be conducted to confirm these findings.

## 4. Materials and Methods

### 4.1. Clinical Examinations

The clinical portion of this study examined the efficacy of an oral hygiene intervention consisting of a SnF_2_ dentifrice, CPC rinse and OR electric toothbrush compared to a basic standard hygiene control [44]. This report includes a post hoc analysis of buccal epithelial samples, which were collected as part of that study, to identify metabolites that are different during the disease process and metabolites that respond to an oral hygiene intervention.

#### 4.1.1. Clinical Design [44]

The study protocol was approved by the BioSci Research Canada, Ltd. Institutional Review Board (70072011LV). The study was a randomized, parallel group, examiner blind clinical study conducted from September to November 2011 at Bio-Sci Research-Las Vegas Nevada, USA, with 69 participants (35 in the negative control group and 34 in the intervention group). Participants were 39 years old on average, ranging from 20 to 69, and 46% of the subjects were female. Groups were well balanced at baseline, with no statistically significant (*p* ≥ 0.395) differences for demographic characteristics (age, ethnicity, gender). Clinical measurements carried out by a trained, calibrated examiner included evaluation of the Gingival Bleeding Index (GBI [73]) and Modified Gingival Index (MGI [74]). Participants were selected based on detailed entry criteria, including a requirement to have at least 20 bleeding sites at baseline. Participants were balanced for group based on baseline measurements, with GBI index averaging 29.957 and MGI measuring 2.086. The participants were assigned to one of two groups following their baseline stratification and instructed to use their assigned products for a period of 6 weeks. The two groups included an intervention group comprising a 0.454% SnF_2_ dentifrice (Crest^®^ Pro-Health Clinical Gum Protection, The Procter & Gamble Company, Cincinnati, OH, USA), an OR electric toothbrush (Oral-B^®^ Professional Care 1000 with Precision Clean brush head, The Procter & Gamble Company, Cincinnati, OH, USA) and a 0.10% CPC rinse (Crest^®^ Pro-Health Clinical Plaque Control, The Procter & Gamble Company, Cincinnati, OH, USA). The control group included a standard 0.243% NaF dentifrice (Crest^®^ Cavity Protection, The Procter & Gamble Company, Cincinnati, OH, USA) and a soft manual toothbrush (Oral-B^®^ Indicator, The Procter & Gamble Company, Cincinnati, OH, USA). Participants were instructed to carry out their assigned hygiene twice per day throughout the 6-week study period. They returned to the clinic for clinical assessments at Weeks 1, 3 and 6. All sixty-nine participants attended each visit and completed the research. Statistical analysis for the clinical measures followed an analysis of covariance.

#### 4.1.2. Gingival Brush Sampling

Gingival brush samples were taken from the maxillary buccal gingiva at baseline, Week 3 and Week 6 clinical visits. A MasterAmp™ Buccal Brush (Catalog #MB100SP; Epicentre Technologies Corp., Madison, WI, USA) was used to sample gingival areas. Four gingival brush samples were collected, two from the left side of the maxillary arch and the other from the right side of the maxillary arch. Gingival brush samples were collected close to the gingival margin of the buccal sites only (preferably from four adjacent teeth in premolar and molar areas). Subjects rinsed for 30 s with 15 mL of essential oils rinse (Listerine, Kenvue, Summit, NJ, USA) in advance of the sample collection to minimize surface contamination of the sampling area. After the essential oils rinse, subjects rinsed again for 30 s with 20 mL of water. Following rinsing, selected sites were isolated with cotton rolls and gently dried with an air syringe. Two gingival brush samples were taken at each site. The sample was placed in a pre-labeled (subject ID, sample ID, visit, and date) vial. The first sample from each site was used for protein and metabolite analysis, and the second sample for RNA extraction. Metabolites are reported here. For metabolite analysis, each brush head was clipped off with sterile scissors and placed into a 15 mL conical tube with 800 µL Dulbecco’s phosphate-buffered saline (DPBS) containing 1X Halt™ Protease Inhibitor Single-Use Cocktail (Thermo Fisher Scientific Inc., Waltham, MA, USA). All collection tubes were vigorously shaken on a multi-tube vortexer for 15 min at 4 °C to extract soluble metabolites and proteins. Using sterile tweezers, the brush heads were dabbed to the side of the tube to collect as much extract as possible and subsequently discarded. Samples were stored at −80 °C until analysis. The samples were taken out of the freezer, then thawed and extracted by placing the samples on a tube shaker for 30 min at 4 °C. The tubes were centrifuged at 15,000 revolutions per minute (RPM) for 10 min in an Eppendorf Centrifuge 5417R (Eppendorf, Mississauga, ON, Canada) to pellet any debris. The supernatants of the extraction were used for metabolite measurements.

#### 4.1.3. Metabolomic Analysis

A total of 69 subjects participated in the clinical study, with 35 assigned to the control group and 34 to the intervention. Gingival brush samples were collected at baseline, Week 3, and Week 6. There were 32 samples from the control group and 29 from the regimen group at baseline. For metabolic profiling, 14 samples were randomly selected from each group at both baseline and Week 3, corresponding to the same subjects (results shown in Figure 1 and Figure 2). This subset demonstrated similar bleeding site outcomes as the total population in the clinical trial, with each group showing a statistically significant (*p* < 0.001) 23% and 21% greater bleeding site reduction, respectively, for the intervention versus the control at Week 3. The remaining baseline samples (18 from the control group and 15 from the intervention group), along with all Week 6 samples (31 from the control group and 32 from the intervention group), were utilized for targeted metabolite analysis (results shown in Figure 3).

For metabolomic profiling, extracted gingival brush samples of 14 control and 14 intervention groups at baseline and Week 3 were randomly selected and analyzed using Metabolon’s global biochemical profiling platforms (Metabolon, Morrisville, NC, USA) following standard procedures [43]. Briefly, samples (125 μL each) were aliquoted for analysis on the Liquid Chromatography-Mass Spectrometer/Mass Spectrometer and Polar LC platforms. Proprietary software was used to match ions to an in-house library of standards for metabolite identification and quantitation by peak area integration.

#### 4.1.4. Statistical Analyses

All statistical analyses and visualizations were conducted using R (version 4.4.3). Scaled data for 170 biomarkers were provided by Metabolon, Inc. (Morrisville, NC, USA); however, only 131 biomarkers with less than 50% non-detects were included in subsequent analyses.

Normality of the log2-transformed data was assessed using the Shapiro–Wilk test (via the R function shapiro.test). For data that met the normality assumption, independent two-sample *t*-tests (conducted using t.test) were performed to compare Test and Control samples at Week 3. Additionally, paired *t*-tests were utilized to evaluate changes in treated samples by comparing Week 3 measurements to baseline values.

For data failing to meet the normality assumption, a fifth root transformation was applied. If normality remained unattainable, non-parametric alternatives were employed: the Mann–Whitney U test for independent samples and the Wilcoxon signed-rank test (using wilcox.test) for paired samples.

The resulting *p*-values were adjusted using the R function p.adjust with the Benjamini–Hochberg (BH) method to generate q-values and control for the false discovery rate (FDR). Metabolites with an FDR-corrected q-value < 0.05 were declared statistically significant. For hypothesis generation and limited by the small sample size, we also considered metabolites with a nominal (unadjusted) *p*-value < 0.05 as the most prominent candidates for further investigation. All discussed metabolites will report both their raw *p*-value and FDR-corrected q-value. Metabolites with a q-value > 0.05 are presented as prominent candidates for further investigation and are not considered statistically significant. Direct measurement of biomarkers—quantitation of citrulline, ornithine, arginine, deoxycarnitine, malic acid, fumaric acid, and succinic acid—from the extracts of the gingival-brush samples was conducted using gradient hydrophilic interaction liquid chromatography coupled with tandem mass spectrometry (HILIC-MS/MS). Gingival extracts were subject to both HILIC-MS/MS and bicinchoninic acid total protein analysis. For free biomarker analysis, the extracts of the gingival-brush samples were analyzed either directly (50 µL) or diluted 5-fold with dilution solution (50/50 acetonitrile/ultra-pure water with 0.754% formic acid). For total biomarker analysis, the extracts of the gingival-brush samples were first hydrolyzed using 6 N HCl (50 µL of extract with 450 µL of 6N HCl) at 110 °C for 16 h. The hydrolyzed samples were then dried under vacuum at room temperature using a Savant SpeedVac Concentrator (Thermo Fisher Scientific Inc., Waltham, MA, USA) and reconstituted in 1 mL of dilution solution for analysis. The standards and the samples were separated on a Sequant ZIC-HILIC Column (Millipore, 2.1 × 150 mm, 5 μm particles, Thermo Fisher Scientific Inc., Waltham, MA, USA) and detected by a Sciex 6500 Triple Quadrupole Mass Spectrometer (AB Sciex LLC, Marlborough, MA, USA). Analytes and the corresponding stable isotope-labeled internal standards (ISTDs) were monitored by electrospray ionization in either positive mode or negative mode using the selected-reaction-monitoring schemes shown in Table 1. A standard curve was constructed by plotting the signal, defined here as the peak area ratio (peak area analyte/peak area ISTD), for each standard versus the concentration of each analyte for the corresponding standard. The concentration of each analyte in the calibration standards and gingival-brush extract samples was then back-calculated using the generated regression equation. The concentration of protein-bound analyte was calculated as the result of subtracting the concentration of free analyte from the concentration of total analyte. The result was reported as the concentration of analyte, or the result was normalized by the amount of total proteins found in the extract.

### 4.2. In Vitro Examinations

The purpose of these experiments was: (1) to determine if LPS stimulates production of NO and citrulline from monocytes and microphages, mimicking the gingivitis condition; and (2) to evaluate the ability of SnF_2_ (alone) to suppress NO and citrulline production, as indicated from the intervention sample analyses.

#### 4.2.1. OMV Isolation

OMVs were prepared and isolated as previously described [40]. Briefly, *P. gingivalis* (American Type Culture Collection (ATCC) catalog #33277, American Type Culture Collection, Manassas, VA, USA) was cultured in MTGE media (Anaerobic Enrichment Broth, Anaerobe Systems, Morgan Hill, CA, USA), and the medium was collected after 72 h by centrifugation. OMVs were secreted by *P. gingivalis* into the MTGE media. The medium was then filtered through 0.45 μm pore polyvinylidene fluoride membranes to remove cell debris. The volume of the medium was reduced by filtration using a tangential flow filtration (TFF) Minimate TFF System (PALL Life Sciences, Port Washington, NY, USA) with filter capsules of molecular weight cutoff 100 kD at 40 psi. The retentate from the filtration was centrifuged at 140,000× *g* for 1 h at 4 °C using an SW32 swinging bucket rotor on a Beckman XL-100K Ultracentrifuge (Beckman Coulter, Atlanta, GA, USA) to separate the OMV pellet from the supernatant. The pellets were resuspended in DPBS buffer (1X DPBS: Life Technologies, Grand Island, NY, USA) and centrifuged at 200,000× *g* for 1 h at 4 °C (using an SW41 swinging bucket rotor) to yield a standard OMV preparation. The OMVs were resuspended in endotoxin-free water, aliquoted into 0.5 mL Eppendorf tubes, and stored at −80 °C. OMVs were quantified using the Bradford assay to estimate protein amounts. The amount of OMV used in experiments was based on the protein concentrations in the OMV preparation (μg/mL).

#### 4.2.2. Nitric Oxide (NO) Assay

RAW 264.7 macrophages (TIB-71, ATCC, USA) were maintained in Dulbecco’s Modified Eagle Medium (DMEM; Thermo Fisher Scientific, Inc., Waltham, MA, USA) supplemented with 10% fetal bovine serum (S1810-500, Biowest, Nuaillé, France). Nitrite production, an indicator of NO synthesis, was measured in the supernatant of RAW 264.7 macrophages. The experiment was repeated 3 times, each with 3 replicates. The cells were cultured in 96-well plates with 100 μL of culture medium until cells reached confluence (approximately 100,000 cells per well). To induce *iNOS*, fresh culture medium containing 500 ng/mL LPS (L-9143, Sigma-Aldrich Co., St. Louis, MO, USA) was added. To assay the inhibition effects of stannous chloride and SnF_2_ on NO production, each was added at a dose range of 15–2500 μM with OMV. The protocol was adapted from Imayoshi et al. [75]. Nitrite and nitrate accumulation in the medium was measured at 24 h post-application. Nitrite in culture medium was reduced to nitrogen dioxide with 0.25 IU/mL nitrate reductase (N7265, Sigma, USA) and 200 μM Nicotinamide Adenine Dinucleotide Phosphate (N6132, Sigma, USA) at room temperature for 20 min. 2, 3-Diaminonapthhalene (DAN) (88461, Sigma, USA), a fluorescent substrate primarily used as a reagent in analytical chemistry for detecting and quantifying nitrite, was dissolved in 0.62 M HCl and added at 250 μg/mL to each well, and placed on a plate shaker @500 RPM for 15 min, protected from light. DAN, when in the presence of nitrogen dioxide, is converted to 2, 3-naphthotriazole (NAT), a fluorescent product. Sodium hydroxide (2.8 N) was added to each well to stop the reaction. Fluorescence was measured using Biotek Cytation 3 multimode reader (Agilent, Santa Clara, CA, USA) with Gen5 v3.14.03 at (Ex:365 nm/Em:450 nm). The amount of nitrogen dioxide in samples was based on a standard curve using Nitrite Ion Standard Solution nitrogen dioxide (140-06451, Fujifilm Wako, Osaka, Japan). Cells were lysed in RIPA buffer (89901, Pierce, Thermo Scientific, USA), and protein concentration was determined via Bradford assay (Coomassie plus Bradford assay kit, 23236, Thermo Scientific, USA) according to the manufacturer’s protocol.

#### 4.2.3. Citrulline Production in Human Primary Blood Monocytes

Human peripheral blood monocytes were purchased and maintained following the vendor’s instructions (StemCell Technologies, Vancouver, BC, Canada). Cells were seeded in a 6-well plate at 200,000 cells/well in 1.5 mL of medium (DMEM + glutaGRO (ThermoFisher Scientific, Waltham, MA, USA)) supplemented with 9.1% fetal bovine serum and 1% penicillin/streptomycin, and treated with LPS 100 ng/mL in the presence or absence of various concentrations of SnF_2_ for 24 h. Monocytes from 4 individuals were used in duplicate in the assay. Citrulline in monocytes was measured using an ELISA kit following the manufacturer’s instructions (CUSABIO, Wuhan, China).

#### 4.2.4. Statistical Analyses

Results of the direct measurement of metabolite biomarkers, NO production assay and citrulline production assay were analyzed using R (version 4.5.1) with the lme4 (version 2.0-1) package for mixed-effects modeling, lmerTest (version 3.2-1) for approximate *p*-value calculation, emmeans (version 2.0.3) for post hoc comparisons, and ggplot2 (version 4.0.3) ggpubr (version 0.6.3) for plots.

To account for the experimental design involving multiple, independent experiments and the inherent variability between experiments, linear mixed-effects models (LMEMs) were employed. In all LMEMs, ‘Experiment’ was included as a random intercept to model within-experiment correlation and between-experiment variability.

Each LMEM investigated a specific dependent variable (e.g., ‘Citrulline concentration’, ‘NO production’, ‘Total cellular protein’) as a function of a fixed effect factor, which included either Concentrations of OMVs, Concentrations of SnF_2_, or Specific treatment conditions (e.g., medium vs. LPS treatments).

Following each LMEM, estimated marginal means (emmeans) were calculated for each group of the respective fixed effect factor. All post hoc comparisons were then performed using emmeans, with *p*-values adjusted using the BH method to control the FDR across the family of comparisons for each model. Model diagnostics (residual distribution, variance homogeneity, and random-effect singularity) were reviewed to assess model adequacy.

## 5. Conclusions

This research assessed metabolite levels in gingival brush samples from study participants with gingivitis who used an oral hygiene intervention (SnF_2_ dentifrice, CPC rinse, and OR electric toothbrush) or control (NaF dentifrice and manual toothbrush). Citrulline was identified as a potential gingivitis marker and increased NO production as a possible driver of oxidative stress. Both the use of the three-product intervention and subsequent in vitro testing of SnF_2_ alone resulted in suppressed NO and oxidative stress levels. This indicates a plausible mechanism involving SnF_2_ for the gingival health benefits of the intervention. Larger-scale studies evaluating the effects of SnF_2_ toothpaste alone, along with research assessing the effect of the toothbrush and rinse used in the intervention, should be assessed in future research.

## Figures and Tables

**Table 1:** Multiple Reaction Monitoring (MRM) transitions for analytes and their corresponding stable isotope labeled internal standards.

Analytes	MRM	Internal Standards	MRM
Citrulline	176 → 159	d_7_-Citrulline	181 → 164
Ornithine	133 → 70	d_6_-Ornithine	139 → 76
Arginine	175 → 70	^13^C_6_-Arginine	181 → 74
Deoxycarnitine	146 → 60	d_9_-Deoxycarnitine	155 → 69
Malic Acid	133 → 115	d_3_-Malic Acid	136 → 117
Fumaric Acid	115 → 71	^13^C_4_-Fumaric Acid	119 → 74
Succinic Acid	117 → 73	^13^C_4_-Succinic Acid	121 → 76

## Data Availability

The datasets generated and analyzed during the current study are not publicly available, as they are considered proprietary, but they may be available from the corresponding author on reasonable request.

## Supplementary Materials

The following supporting information can be downloaded at: https://www.mdpi.com/article/10.3390/ijms27135882/s1.

## Abbreviations

The following abbreviations are used in this manuscript:

9-HODE	9-hydroxyoctadecadienoic acid
13-HODE	13-hydroxyoctadecadienoic acid
ATCC	American Type Culture Collection
ATP	Adenosine Triphosphate
BH	Benjamini-Hochberg
CPC	Cetylpyridinium Chloride
DAN	2,3-Diaminonapthhalene
DMEM	Dulbecco’s Modified Eagle Medium
DPBS	Dulbecco’s Phosphate-Buffered Saline
ELISA	Enzyme-Linked Immunosorbent Assay
FDR	False Discovery Rate
GBI	Gingival Bleeding Index
HILIC-MS/MS	Hydrophilic Interaction Liquid Chromatography Coupled with Tandem Mass Spectrometry
*ICAM-1*	Intracellular Adhesion Molecule 1
*iNOS*	Inducible Nitric Oxide Synthase
ISTDs	Internal Standards
LMEM	Linear Mixed-Effects Model
LPS	Lipopolysaccharide
LTA	Lipoteichoic Acid
MGI	Modified Gingival Index
MRM	Multiple Reaction Monitoring
NAT	2,3-Naphthotriazole
NO	Nitric Oxide
OMVs	Outer Membrane Vesicles
OR	Oscillating-Rotating
PAMPs	Pathogen-Associated Molecular Patterns
PRRs	Pattern Recognition Receptors
RPM	Revolutions Per Minute
SnF_2_	Stannous fluoride
TFF	Tangential Flow Filtration
*TLR*s	Toll-like Receptors
*VCAM-1*	Vascular Cell Adhesion Molecule 1
*VEGF*	Vascular Endothelial Growth Factor

## References

[1] Stamm J.W. (1986). Epidemiology of gingivitis. J. Clin. Periodontol..

[2] Nazir M.A. (2017). Prevalence of periodontal disease, its association with systemic diseases and prevention. Int. J. Health Sci..

[3] Nazir M., Al-Ansari A., Al-Khalifa K., Alhareky M., Gaffar B., Almas K. (2020). Global prevalence of periodontal disease and lack of its surveillance. Sci. World J..

[4] Bamashmous S., Kotsakis G.A., Kerns K.A., Leroux B.G., Zenobia C., Chen D., Trivedi H.M., McLean J.S., Darveau R.P. (2021). Human variation in gingival inflammation. Proc. Natl. Acad. Sci. USA.

[5] Kassebaum N.J., Bernabé E., Dahiya M., Bhandari B., Murray C.J., Marcenes W. (2014). Global burden of severe periodontitis in 1990–2010: A systematic review and meta-regression. J. Dent. Res..

[6] Dye B.A. (2012). Global periodontal disease epidemiology. Periodontol. 2000.

[7] Petersen P.E., Ogawa H. (2012). The global burden of periodontal disease: Towards integration with chronic disease prevention and control. Periodontol. 2000.

[8] Lang N.P., Schätzle M.A., Löe H. (2009). Gingivitis as a risk factor in periodontal disease. J. Clin. Periodontol..

[9] Schätzle M., Löe H., Bürgin W., Anerud A., Boysen H., Lang N.P. (2003). Clinical course of chronic periodontitis. I. Role of gingivitis. J. Clin. Periodontol..

[10] Schätzle M., Löe H., Lang N.P., Bürgin W., Anerud A., Boysen H. (2004). The clinical course of chronic periodontitis. J. Clin. Periodontol..

[11] Lang N.P., Adler R., Joss A., Nyman S. (1990). Absence of bleeding on probing. An indicator of periodontal stability. J. Clin. Periodontol..

[12] Joss A., Adler R., Lang N.P. (1994). Bleeding on probing. A parameter for monitoring periodontal conditions in clinical practice. J. Clin. Periodontol..

[13] Liu W., Cao Y., Dong L., Zhu Y., Wu Y., Lv Z., Iheozor-Ejiofor Z., Li C. (2019). Periodontal therapy for primary or secondary prevention of cardiovascular disease in people with periodontitis. Cochrane Database Syst. Rev..

[14] de Molon R.S., Rossa C., Thurlings R.M., Cirelli J.A., Koenders M.I. (2019). Linkage of periodontitis and rheumatoid arthritis: Current evidence and potential biological interactions. Int. J. Mol. Sci..

[15] Kaur S., White S., Bartold M. (2012). Periodontal disease as a risk factor for rheumatoid arthritis: A Systematic Review. JBI Libr. Syst. Rev..

[16] Hajishengallis G., Chavakis T. (2021). Local and systemic mechanisms linking periodontal disease and inflammatory comorbidities. Nat. Rev. Immunol..

[17] Kapila Y.L. (2021). Oral health’s inextricable connection to systemic health: Special populations bring to bear multimodal relationships and factors connecting periodontal disease to systemic diseases and conditions. Periodontol. 2000.

[18] Madianos P.N., Bobetsis Y.A., Kinane D.F. (2005). Generation of inflammatory stimuli: How bacteria set up inflammatory responses in the gingiva. J. Clin. Periodontol..

[19] Holt S.C., Bramanti T.E. (1991). Factors in virulence expression and their role in periodontal disease pathogenesis. Crit. Rev. Oral Biol. Med..

[20] Imamura T. (2003). The role of gingipains in the pathogenesis of periodontal disease. J. Periodontol..

[21] Morita M., Wang H.L. (2001). Relationship of sulcular sulfide level to severity of periodontal disease and BANA test. J. Periodontol..

[22] Niederman R., Zhang J., Kashket S. (1997). Short-chain carboxylic-acid-stimulated, PMN-mediated gingival inflammation. Crit. Rev. Oral Biol. Med..

[23] Hamada S., Takada H., Ogawa T., Fujiwara T., Mihara J. (1990). Lipopolysaccharides of oral anaerobes associated with chronic inflammation: Chemical and immunomodulating properties. Int. Rev. Immunol..

[24] Ginsburg I. (2002). Role of lipoteichoic acid in infection and inflammation. Lancet Infect. Dis..

[25] Zhang Z., Liu D., Liu S., Zhang S., Pan Y. (2021). The Role of *Porphyromonas gingivalis* Outer Membrane Vesicles in Periodontal Disease and Related Systemic Diseases. Front. Cell. Infect. Microbiol..

[26] Beutler B., Jiang Z., Georgel P., Crozat K., Croker B., Rutschmann S., Du X., Hoebe K. (2006). Genetic analysis of host resistance: Toll-like receptor signaling and immunity at large. Annu. Rev. Immunol..

[27] Takeda K., Akira S. (2005). Toll-like receptors in innate immunity. Int. Immunol..

[28] Hans M., Hans V.M. (2011). Toll-like receptors and their dual role in periodontitis: A review. J. Oral Sci..

[29] Klukowska M., Haught J.C., Xie S., Circello B., Tansky C.S., Khambe D., Huggins T., White D.J. (2017). Clinical Effects of Stabilized Stannous Fluoride Dentifrice in Reducing Plaque Microbial Virulence I: Microbiological and Receptor Cell Findings. J. Clin. Dent..

[30] Xie S., Haught J.C., Tansky C.S., Klukowska M., Hu P., Ramsey D.L., Circello B., Huggins T.G., White D.J. (2018). Clinical effects of stannous fluoride dentifrice in reducing plaque microbial virulence III: Lipopolysaccharide and TLR2 reporter cell gene activation. Am. J. Dent..

[31] Konradsson K., Lingström P., Emilson C.G., Johannsen G., Ramberg P., Johannsen A. (2020). Stabilized stannous fluoride dentifrice in relation to dental caries, dental erosion and dentin hypersensitivity: A systematic review. Am. J. Dent..

[32] He T., Zou Y., DiGennaro J., Biesbrock A.R. (2022). Novel findings on anti-plaque effects of stannous fluoride. Am. J. Dent..

[33] Biesbrock A., He T., DiGennaro J., Zou Y., Ramsey D., Garcia-Godoy F. (2019). The effects of bioavailable gluconate chelated stannous fluoride dentifrice on gingival bleeding: Meta-analysis of eighteen randomized controlled trials. J. Clin. Periodontol..

[34] Hu D., Li X., Liu H., Mateo L.R., Sabharwal A., Xu G., Szewczyk G., Ryan M., Zhang Y.-P. (2019). Evaluation of a stabilized stannous fluoride dentifrice on dental plaque and gingivitis in a randomized controlled trial with 6-month follow-up. J. Am. Dent. Assoc..

[35] Parkinson C.R., Amini P., Jose A., Gallob J. (2018). A 12-week randomized clinical study investigating the anti-gingivitis efficacy of a 0.454% w/w stannous fluoride dentifrice. Am. J. Dent..

[36] Cannon M., Khambe D., Klukowska M., Ramsey D.L., Miner M., Huggins T., White D.J. (2018). Clinical effects of stabilized stannous fluoride dentifrice in reducing plaque microbial virulence II: Metabonomic changes. J. Clin. Dent..

[37] Gumber H.K., Louyakis A.S., Sarma T., Fabijanic K.I., Paul R., Mellenbruch K., Kilpatrick-Liverman L. (2022). Effect of a stannous fluoride dentifrice on biofilm composition, gene expression and biomechanical properties. Microorganisms.

[38] Haught J.C., Xie S., Circello B., Tansky C.S., Khambe D., Sun Y., Lin Y., Sreekrishna K., Klukowska M., Huggins T. (2016). Lipopolysaccharide and lipoteichoic acid binding by antimicrobials used in oral care formulations. Am. J. Dent..

[39] Haught C., Xie S., Circello B., Tansky C.S., Khambe D., Klukowska M., Huggins T., White D.J. (2016). Lipopolysaccharide and Lipoteichoic Acid Virulence Deactivation by Stannous Fluoride. J. Clin. Dent..

[40] Xie S., Tansky C.S., Ashe J., Gao F., Ramji N.B., Iberi V., Sun Y., Ramji N., Biesbrock A.R. (2024). Stannous fluoride protects gingival keratinocytes against infection and oxidative stress by *Porphyromonas gingivalis* outer membrane vesicles. Front. Dent. Med..

[41] Klukowska M.A., Ramji N., Combs C., Milleman J.L., Milleman K.R., Ramsey D.L., Rattanaudompol U., Haven C., McClenathan D., White D.J. (2018). Subgingival uptake and retention of stannous fluoride from dentifrice: Gingival crevicular fluid concentrations in sulci post-brushing. Am. J. Dent..

[42] Xie S., Klukowska M., Wang J., Huggins T., Ashe J., Tansky C.S., Li L., Circello B., Ramji N., White D.J. (2026). Gingivitis pathogenesis involves upregulation of glycolysis and citric acid cycle activity mediated by bacterial virulence. Int. J. Mol. Sec..

[43] Ryals J., Lawton K., Stevens D., Milburn M. (2007). Metabolon, Inc. Pharmacogenomics.

[44] Klukowska M., Goyal C.R., Barker M.L., Hoke P., Qaqish J., Conde E. Antigingivitis efficacy of combined antimicrobial and mechanical oral hygiene. Proceedings of the 2013 IADR/AADR/CADR 91st General Session.

[45] Palmieri E.M., McGinity C., Wink D.A., McVicar D.W. (2020). Nitric Oxide in Macrophage Immunometabolism: Hiding in Plain Sight. Metabolites.

[46] Takashiba S., Van Dyke T.E., Amar S., Murayama Y., Soskolne A.W., Shapira L. (1999). Differentiation of monocytes to macrophages primes cells for lipopolysaccharide stimulation via accumulation of cytoplasmic nuclear factor kappaB. Infect. Immun..

[47] Cinelli M.A., Do H.T., Miley G.P., Silverman R.B. (2020). Inducible nitric oxide synthase: Regulation, structure, and inhibition. Med. Res. Rev..

[48] Facchin B.M., Dos Reis G.O., Vieira G.N., Mohr E.T.B., da Rosa J.S., Kretzer I.F., Demarchi I.G., Dalmarco E.M. (2022). Inflammatory biomarkers on an LPS-induced RAW 264.7 cell model: A systematic review and meta-analysis. Inflamm. Res..

[49] Lee C.I., Liu X., Zweier J.L. (2000). Regulation of xanthine oxidase by nitric oxide and peroxynitrite. J. Biol. Chem..

[50] Ionazzi A., Giangregorio N., Console L., De Palma A., Indiveri C. (2017). Nitric oxide inhibits the mitochondrial carnitine/acylcarnitine carrier through reversible S-nitrosylation of cysteine 136. Biochim. Biophys. Acta Bioenerg..

[51] Kaduce T.L., Figard P.H., Leifur R., Spector A.A. (1989). Formation of 9-hydroxyoctadecadienoic acid from linoleic acid in endothelial cells. J. Biol. Chem..

[52] Vangaveti V., Baune B.T., Kennedy R.I. (2010). Hydroxyoctadecadienoic acids: Novel regulators of macrophage differentiation and atherogenesis. Ther. Adv. Endocrinol. Metab..

[53] Szczuko M., Kotlega D., Palma J., Zembron-Lacny A., Tylutka A., Golab-Janowska M., Drozd A. (2020). Lipoxins, RevD1 and 9, 13 HODE as the most important derivatives after an early incident of ischemic stroke. Sci. Rep..

[54] Tsuchida K., Sakiyama N. (2023). 9-Hydroxyoctadecadienoic acid plays a crucial role in human skin photoaging. Biochem. Biophys. Res. Commun..

[55] Friederichs B., Toborek M., Hennig B., Heinevetter L., Muller C., Brigelius-Flohe R. (1999). 13-HPODE and 13-HODE modulate cytokine-induced expression of endothelial cell adhesion molecules differently. Biofactors.

[56] Tannahill G.M., Curtis A.M., Adamik J., Palsson-McDermott E.M., McGettrick A.F., Goel G., Frezza C., Bernard N.J., Kelly B., Foley N.H. (2013). Succinate is an inflammatory signal that induces IL-1β through HIF-1α. Nature.

[57] Wu K.K. (2023). Extracellular Succinate: A Physiological Messenger and a Pathological Trigger. Int. J. Mol. Sci..

[58] Connors J., Dawe N., Van Limbergen J. (2019). The role of succinate in the regulation of intestinal inflammation. Nutrients.

[59] Guha M., Mackman N. (2001). LPS induction of gene expression in human monocytes. Cell. Signal..

[60] Orecchioni M., Ghosheh Y., Pramod A.B., Ley K. (2019). Macrophage Polarization: Different Gene Signatures in M1(LPS+) vs. Classically and M2(LPS-) vs. Alternatively Activated Macrophages. Front. Immunol..

[61] Bosshart H., Heinzelmann M. (2007). Targeting bacterial endotoxin: Two sides of a coin. Ann. N. Y. Acad. Sci..

[62] Apte R.S., Chen D.S., Ferrara N. (2019). VEGF in Signaling and Disease: Beyond Discovery and Development. Cell.

[63] Gölz L., Memmert S., Rath-Deschner B., Jäger A., Appel T., Baumgarten G., Götz W., Frede S. (2015). Hypoxia and *P. gingivalis* synergistically induce HIF-1 and NF-κB activation in PDL cells and periodontal diseases. Mediat. Inflamm..

[64] Kinane D.F., Adonogianaki E., Moughal N., Winstanley F.P., Mooney J., Thornhill M. (1991). Immunocytochemical characterization of cellular infiltrate, related endothelial changes and determination of GCF acute-phase proteins during human experimental gingivitis. J. Periodontal Res..

[65] Moughal N.A., Adonogianaki E., Thornhill M.H., Kinane D.F. (1992). Endothelial cell leukocyte adhesion molecule-1 (ELAM-1) and intercellular adhesion molecule-1 (ICAM-1) expression in gingival tissue during health and experimentally-induced gingivitis. J. Periodontal Res..

[66] Samaranayake L., Matsubara V.H. (2017). Normal Oral Flora and the Oral Ecosystem. Dent. Clin. N. Am..

[67] Sudhakara P., Gupta A., Bhardwaj A., Wilson A. (2018). Oral Dysbiotic Communities and Their Implications in Systemic Diseases. Dent. J..

[68] Gao L., Xu T., Huang G., Jiang S., Gu Y., Chen F. (2018). Oral microbiomes: More and more importance in oral cavity and whole body. Protein Cell.

[69] Siqueira J.F., Rôças I.N. (2017). The Oral Microbiota in Health and Disease: An Overview of Molecular Findings. Methods Mol. Biol..

[70] Wagner-Golbs A., Neuber S., Kamlage B., Christiansen N., Bethan B., Rennefahrt U., Schatz P., Lind L. (2019). Effects of Long-Term Storage at -80 °C on the Human Plasma Metabolome. Metabolites.

[71] Sugimoto M., Saruta J., Matsuki C., To M., Onuma H., Kaneko M., Soga T., Tomita M., Tsukinoki K. (2013). Physiological and environmental parameters associated with mass spectrometry-based salivary metabolomic profiles. Metabolomics.

[72] Gardner A., Carpenter G., So P.W. (2020). Salivary metabolomics: From diagnostic biomarker discovery to investigating biologic function. Metabolites.

[73] Saxton C.A., van der Ouderaa F.J. (1989). Effects of a dentifrice containing zinc citrate and triclosan on developing gingivitis. J. Periodontal Res..

[74] Lobene R., Weatherford T., Ross N., Lamm R.A., Menaker L. (1986). A modified gingival index for use in clinical trials. Clin. Prev. Dent..

[75] Imayoshi R., Cho T., Kaminishi H. (2011). NO production in RAW264 cells stimulated with *Porphyromonas gingivalis* extracellular vesicles. Oral Dis..

