# Supplementary material for: Effects of Oral Care Intervention on Gingivitis-Mediated Induction of Nitric Oxide Synthesis and Oxidative Stress
Source: Int J Mol Sci. 2026 Jun 30;27(13):5882. doi: 10.3390/ijms27135882 (PMC13361661; doi:10.3390/ijms27135882)
Supplement: Supplementary file 1 [file ijms-27-05882-s001.zip › Supplementary Table 1 May 2026.pdf]

Supplementary Table S1. Between-group comparisons for gingivitis efficacy endpoints using Analysis of Covariance.

	Number of Gingival Bleeding Sites			Modified Gingival Index Score		
Visit/Treatment	Adjusted Mean (SE)	% Reduction vs. Control	p-value	Adjusted Mean (SE)	% Reduction vs. Control	p-value
Week 1						
Negative Control	27.930 (0.126)		<0.0001	2.053 (0.003)		<0.0001
Intervention	25.425 (0.209)	9.0%		2.015 (0.004)	1.9%	
Week 3						
Negative Control	25.944 (0.296)		<0.0001	2.026 (0.007)		<0.0001
Intervention	20.540 (0.445)	20.8%		1.943 (0.009)	4.1%	
Week 6						
Negative Control	22.895 (0.190)		<0.0001	1.960 (0.007)		<0.0001
Intervention	10.497 (0.825)	54.2%		1.731 (0.013)	11.6%	

SE = Standard Error.
